# Ni ions doped oxyflourophosphate glass as a triple ultraviolet–visible–near infrared broad bandpass optical filter

**DOI:** 10.1038/s41598-022-20300-w

**Published:** 2022-09-26

**Authors:** W. A. Abu-raia, D. A. Aloraini, S. A. El-Khateeb, Aly Saeed

**Affiliations:** 1 Basic Science Department, Institute of Aviation Engineering and Technology, Giza, Egypt; 2 Department of Physics, College of Science, Princess Nourah Bint Abdulrahman University (PNU), Riyadh, Saudi Arabia; 3 Nuclear Medicine Technology Department, Inaya Medical Colleges (IMC), Riyadh, Saudi Arabia; 4 Nuclear Power Stations Department, Faculty of Engineering, Egyptian-Russian University, Cairo, Egypt

**Keywords:** Materials science, Optics and photonics, Physics

## Abstract

Research and development R&D about new materials that can be used as an optical filter, shortpass, bandpass, and longpass is still ongoing. So, in this context, the 50P_2_O_5_–20ZnF_2_–15MgF_2_–15PbF_2_ doped different concentrations of NiO ranging from 0 up to 4 mol% as a bandpass filter was prepared using the conventional melt annealing method. The formation of the amorphous essence was observed in the X-ray diffraction patterns. The role of Ni ions in the produced glass network was studied by density and Fourier Transform Infrared FTIR spectroscopy results, which showed that the Ni ions play a network modifier role. Thermal analysis results showed high thermal stability for the produced glasses. Electron paramagnetic resonance EPR spectra results clarified that the Ni^3+^ ions occupy an elongated octahedral $\left( {g_{ \bot } > g_{\parallel } \approx 2} \right)$. The measurements of AC conductivity, dielectric constant, and electric modulus were studied at different temperatures and frequencies. The ionic conduction dominates the conductivity at high temperatures, while the electronic dominates at low temperatures. The appearance of Ni^3+^ (confirmed by ESR) and Ni^2+^ were observed in the optical absorption spectra, also it was found that both of them occupy both tetrahedral and octahedral sites. Three distinguished bands in the UV (centered at 354 nm), visible (centered at 620 nm), and NIR (centered at 1074 nm) regions appeared in the optical transmittance spectra, indicating the defining characteristic of the bandpass filter.

## Introduction

Controlling the optical transmittance of a specific region of electromagnetic waves requires special character materials. The optical filters; shortpass, bandpass, and longpass are the materials assigned to control the wave spectrum^1–4^. Among them, the bandpass filters have the simplest and most economical role in the transmission of a well-defined band of light and rejecting all unwanted wavelengths^1–4^. This type of filter is a mainstay of many optical applications such as laser line flame photometry, fluorescence applications, UV sterilization, spectral radiometry, chemical analysis, paint-color formulation, machine vision, and biotech instrumentation. Filter materials must have a narrow transmissive band and a particular wavelength region and be a strong deterrent for all other undesirable regions^5,6^.

Glass materials are one of the utilization prevalent materials as optical filters due to their unique properties such as high transparency, thermal stability, and suitable mechanical and thermo-mechanical properties^7,8^. Especially the phosphate glass network, which is a great attractant in this field because it can accommodate high concentrations of active ions without mislaying their properties^7–9^. Fluorides are usually introduced into the glass network as modifiers to adjust some properties such as increasing the optical transmittance, reducing the optical loss in the near and mid-infrared regions, improving thermal stability, and reducing hygroscopic nature^7–9^.

The optical selective properties of the transition metal ions make them an ideal candidate for the bandpass filters^8,9^. Among them is nickel ion that gives different colors to glass ranging from yellow to dark green. Several visible and near-infrared absorption bands in the regions of the divalent state make it rich with optical properties^10–12^. On the other hand, the semiconducting nature of glass inlaid with transition metals ions has made it of unique importance in many optical applications such as lasing media, optical switching devices, and erasable optical recording media^12^. In their manuscript published in 2018^1^, Ashraf M. Emara and El Sayed Youssef studied two blends of phosphate glass containing nickel in the formula 50P_2_O_5_–30ZnO–20NiO and 50P_2_O_5_–30ZnO–20[NiCl_2_–6H_2_O]. The study of the bandpass optical filter behavior of the considered glass in the region of 190–1100 nm showed that two bands are in the UV region of 311–376 nm and the visible region of 617–684 nm. Y. H. Elbashar et al. in 2020^3^ studied the double bandpass filter behavior for a blend of phosphate glass inlaid with different concentrations of nickel in the optical range of 200–2500 nm. They concluded that the transmittance and the FWMH of the two bands decreased with increasing nickel concentrations. In 2022, Essam B. Moustafa et al. studied the role of Vanadium in forming a bandpass filter in the optical range from 190 to 1500 nm. The authors used the 0.45BaO–0.05Al2O3–0.5P2O5 glass system and doped it with different concentrations of V2O5. They found that the V ion has the ability to form a transmittance bandpass in the ranges of 236–324 nm, 412–482 nm, and 682–1500 nm^13^.

In the present article and based on aspects that are discussed in the introduction, an oxyflourophosphate glass inlaid by Ni ions was produced. Ni ions' impact on the oxyflourophosphate glass network was structurally studied through X-ray diffraction, density, and FTIR; thermally through differential scanning calorimeter DSC; magnetically through electron paramagnetic resonance EPR; and electrically through ac conductivity and dielectric properties. UV–visible–NIR spectra were measured and extensively studied to explore the Ni ions' oxidation states and filtering behavior of the produced glass. The novelty of the present article is based on the study of the bandpass filter with triple bands in UV, visible, and NIR regions. On the other hand, metal fluorides were used, which are characterized by low phonon energy and high quantum yield instead of their oxides to achieve high transmittance.

## Materials and methods

High purity of (NH_4_)_2_HPO_4_, ZnF_2_, MgF_2_, PbF_2_, and NiO were carefully weighted and well mixed in an agate mortar to produce a glassy system 50P_2_O_5_–20ZnF_2_–15MgF_2_–15PbF_2_ doped with a 1, 2, 3, and 4 mol % of NiO. The synthesized mixture was meted at 1000 °C for 1 h. with continuous shaking to ensure high homogeneity and then annealed at 250 °C for 1 h. to remove any residual stress. The XRD-Shimadzu diffractometer at room temperature was used to check the physical nature of the produced materials. Density was measured using Archimedes' rule and its results were also used to calculate the molar volume and mean phosphor–phosphor separation^2,14,15^. The spectral range 4000–400 cm^−1^ of Fourier transform computerized infrared spectrometer type (JASCO, FT/IR-6100, Japan) was used to study the building units of the fabricated glasses. A differential scanning calorimeter DSC was used at a heating rate of 5/min up to 600 °C in a high purity nitrogen atmosphere at a flow rate of 15 Psi to measure the glass transition $T_{g}$ and the onset of crystallization $T_{C}$ temperatures. EMX-Bruker ESR spectrometer system equipped with 100 GHz field modulation was used to measure the ESR spectra of the produced materials. An automatic programmable LCR meter was used to examine the AC measurements as a function in both wide ranges of frequency and temperature. Samples were prepared for measurements by polishing them to obtain a smooth, uniform texture, then coated with silver paste. Absorptions and transmission spectra were conducted in the range 190 – 2200 nm on smooth highly polished samples with thicknesses varied from 1.72 to 1.88 mm using JASCO Model V-570 UV–VIS–NIR spectrophotometer.

## Results and discussion

**Figure 1:**
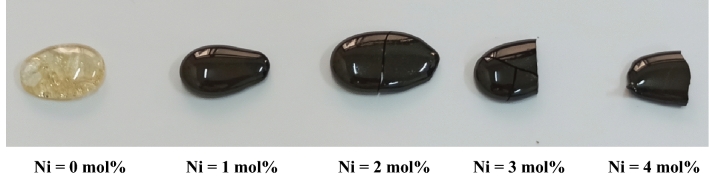
Part of the produced glasses.

Nickel ion enriches glass with a color gradation as it is a transitional metal element, which the color arises due to Ni^2+^ and/or Ni^3+^. The ocular inspection of the considered glasses, which depicted in Fig. 1 showed that the un-doped Ni sample has a light yellow color arises due to the Pb^2+^ ion. Nickel imparts a dark brown color to the considered glass network, which resulted due to the formation of six coordinated Ni (NiO_6_) within the glass network.

### X-ray diffraction, XRD

**Figure 2:**
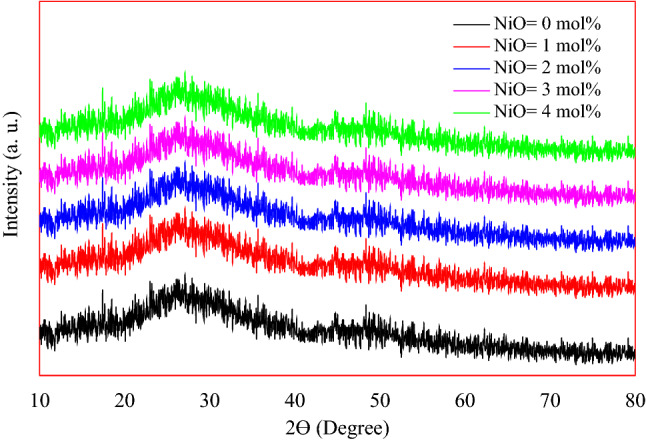
XRD diffraction pattern of the studied materials.

The absence of sharp Bragg’s peak in the X-ray diffraction spectra as shown in Fig. 2 proven no crystallinity in the produced materials. No change was observed in the X-ray diffraction patterns with the increase of Ni concentrations, which means that the amorphous state is maintained with the change in the chemical composition.

### Density

**Figure 3:**
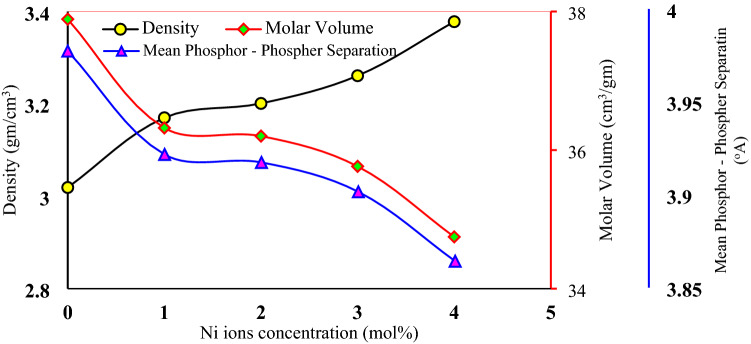
Density, molar volume, and mean phosphor—phosphor of the studied samples.

The insertion of nickel ions led to an increase in the density of the glass and a decrease in its molar volume as shown in Fig. 3. This behavior is attributed that the nickel ions penetrating the interstitial positions of the glass network causing a convergence of the free spaces of the network of the studied glass, which means its compactness. The decreasing behavior of the mean phosphor—phosphor separation as shown in Fig. 3 confirmed this compactness.

### FTIR

**Figure 4:**
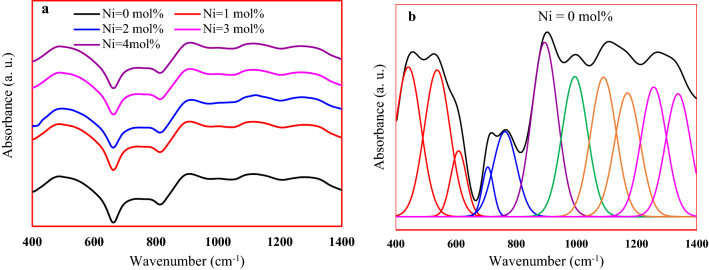
(**a**) FTIR spectra and (**b**) the deconvlision of the studied sample.

The FTIR spectra of all glass samples and deconvolution of the Ni-free sample as an example are shown in Fig. 4. In the Ni-free sample, eleven resolved bands appeared in the deconvoluted spectrum, Fig. 4b.

The band located at 441 cm^-1^ belongs to O–P–O bending vibration in Q^1^ group or perhaps to $\delta \left( {PO_{2} } \right)$ lattice mode vibrations of $\left( {{\text{PO}}_{2}^{ - } } \right)_{{\text{n}}}$ of chain groups^8,16,17^. The apparent band at 537 cm^-1^ is ascribed to O = P–O bending vibration or it may be a fundamental frequency of ${\text{PO}}_{4}^{ - 3}$^2,8,16^. Starting from 2 mol% of NiO, the above two bands shifted towards a higher wavenumber suggesting the phosphate network depolymerization. The bending vibration of P = O units is observed at 615 cm^-1^^16^. The symmetric $\upsilon_{s} \left( {{\text{P}} - {\text{O}} - {\text{P}}} \right)$ stretching modes of bridging oxygen atoms linked with a phosphorus atom in a Q^2^ phosphate tetrahedron are observed at 706, while that of asymmetric $\upsilon_{as} \left( {{\text{P}} - {\text{O}} - {\text{P}}} \right)$ at 762 cm^-1^ and 894 cm^-1^^2,8,16–18^. The band at 995 cm^-1^ is assigned to the symmetric stretching of $\upsilon_{s} \left( {PO_{3} } \right)^{ - 2}$ and that at 1090 cm^-1^ is to the asymmetric stretching of $\upsilon_{as} \left( {PO_{3} } \right)^{ - 2}$ modes of chain-terminating Q^1^ groups^2,8,16–18^. The band at 1170 cm^-1^ is allocated to the symmetric stretching of ${\text{PO}}_{2}^{ - }$ groups^8,16,17^. The band at 1260 cm^-1^ is attributed to the stretching of the doubly bonded oxygen vibration, $\upsilon_{as}$ (P = O) modes, while that at 1327 cm^-1^ is related to the harmonics of those modes^16,17^. The entry and growing of the nickel ions did not lead to the emergence of any other bands, which confirms its role as a modifier to the studied glass network. This role has also appeared through the affected of the positions, broadening, and intensity of absorption bands due to the penetration of Ni ions contents in the glass network.

### Thermal properties

**Figure 5:**
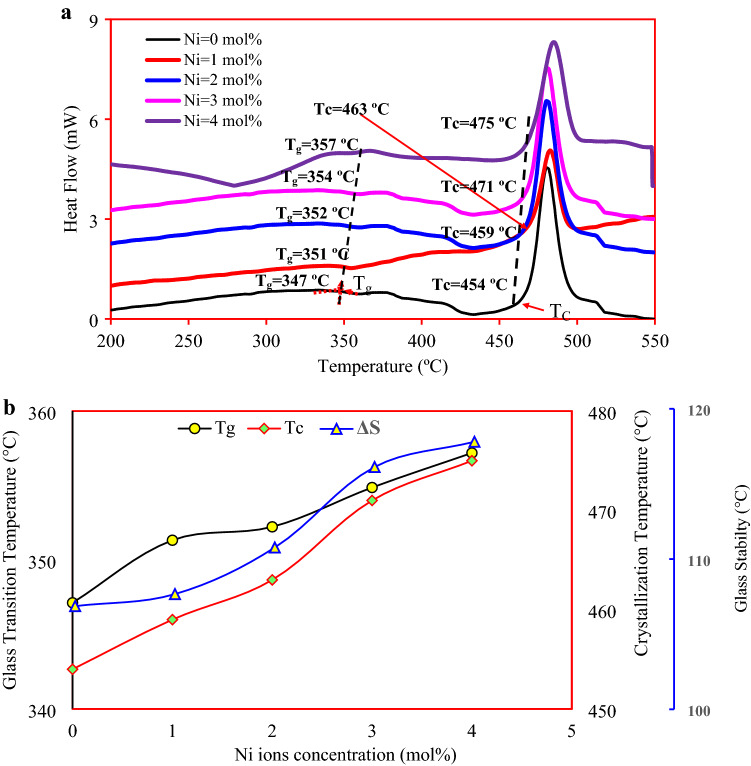
(**a**) DSC thermograms (**b**) Variation of glass transition temperature $T_{g}$, crystallization temperature $T_{C}$, and thermal stability $\Delta S$.

Figure 5a shows the thermal profile obtained from DSC measurements. The characteristic peak of the glass transition temperature is observed in the region from 347 up to 357 °C, while a strong exothermic peak characterizing the crystallization appeared in the region from 463 up to 475 °C. Figure 5b shows the change in the glass transition temperature $T_{g }$, onset crystallization temperature $T_{c }$, and thermal stability $\Delta S \left( {\Delta S = T_{c} - T_{g} } \right)$^19^ with growing nickel ions concentrations. The augmentation of the glass transition temperature is attributed to the tight packing structure caused by the combination of Ni ions in the blended oxyflourophosphate network. This behavior indicates the convergence in the glass network, which was clearly shown in the behavior of density, molar volume, and mean phosphor—phosphor separation. Ni ions’ inclusion in the blended network improves the glass stability and the further increase of Ni drastically enhanced the stability.

### EPR

**Figure 6:**
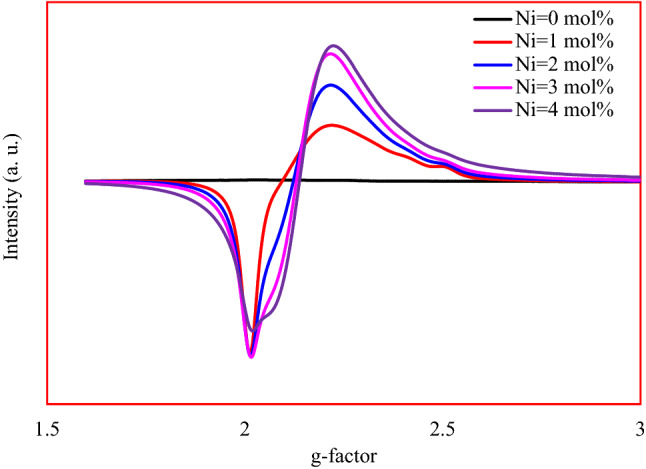
EPR spectra of the studied glasses.

No signal in EPR spectrum of the Ni-free sample was detected as shown in Fig. 6, which indicates the absence of paramagnetic impurities in the base glass samples. Introducing Ni ions generate only one broadband resonance signal. For 3d^8^ of Ni^2+^ and 3d^7^ of Ni^3+^, the g-factor ranged from 2.3 to 2.1. Generally, Ni^2+^ state is undetectable in EPR at room temperature due to spin–orbit coupling of orbitally nondegenerate ground states. Another reason for the absence of Ni^2+^ EPR signal is its strong axial distortion of the octahedral crystal field^20,21^. Two possible probabilities, compressed and elongated octahedral for Ni^3+^ can be observed in EPR. In the present study, $g_{ \bot } > g_{\parallel } \approx 2$ corresponding to elongated octahedral. Here, the $d_{xy} , d_{xz} , d_{yz}$ are represented to the ground state and the transitions conducted as $d_{xz} \to d_{{z^{2} }}$ and $d_{yz} \to d_{{z^{2} }}$. The obtained value of $\overline{g} = 1/3\left( {g_{\parallel } + 2g_{ \bot } } \right)$ closed to 2 not 13/3, so the ground state should be low spin ($s = 1/2$) ^2^E (t_2_^6^e). The observed increase in EPR single with the Ni^3+^ increase is attributed to the ability of Ni^3+^ to form clusters. It is worth noting that Ni^1+^ (3d^9^), which has typical trigonal bipyramidal complexes, cannot be distinguished from Ni^3+^ (3d^7^) because it also has $g_{ \bot } > g_{\parallel } \approx 2$.

### Electrical conductivity

**Figure 7:**
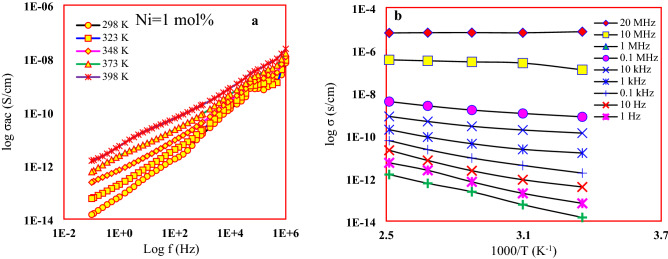
Ac conductivity dependence on (**a**) Frequency and (**b**) Temperature for the considered glasses.

A conductivity dependence on frequency and temperature, 1 mol of Ni as an example, is shown in Fig. 7. The other samples have the same tendencies. In general, the amorphous semiconducting materials obey a Jonscher’s universal power law^22–24^

$$\sigma^{^{\prime}} \left( \omega \right) = \sigma_{dc} + A\omega^{s}$$

$\sigma_{dc}$ is a dc conductivity, A is a constant that gives a polarizability strength, $\omega$ is the angular frequency, and $s$ is the frequency exponent $\left( {0.5 < s < 1} \right)$ and studies the interaction degree between the mobile ions.

According to Fig. 7, the ac conductivity is strongly dependent on frequency in both low and high frequencies and perfectly matched with Jonscher’s universal power law. The results showed an almost invariably augmentation in conductivity with the rising in both frequency and temperature, which was attributed to the concentration of mobility or hopping charge carriers. It was also observed that the dependence of the conductivity on the temperature in the low-frequency region is higher than in the high frequencies, which refers to the dominance of the ionic conduction at high temperatures and the electronic at low temperatures. The semiconducting nature of the studied glass was clearly observed through the increase in conductivity with ascending temperature.

**Figure 8:**
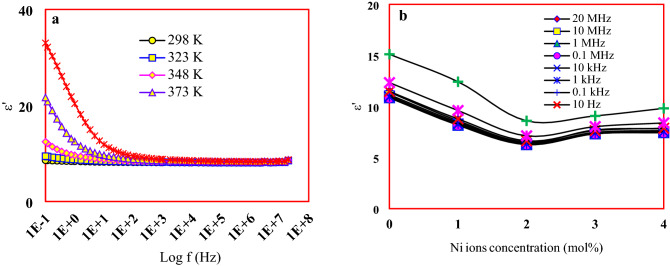
(**a**) Dielectric constant dependence on temperature and frequency and (**b**) Variation of dielectric constant with Ni contents.

A high value of the dielectric constant in the low-frequency region followed by a noticeable decrease until reaching the constancy in the high-frequency region for different temperatures is observed in Fig. 8a. The high values of dielectric constant in the low-frequency region result from the occurrence of rapid polarization within the studied glass network. In the low-frequency region, a higher value of the dielectric constant means that there is an obstacle that does not allow the charges to pass and move to the external circuit, which means piling up of charges on the interface between the electrode and the sample. And then a large polarization occurs in the material as a result of the accumulation of ions near the electrode. On the other hand, the reduction in the dielectric constant with the increase of frequency arises due to the effect of ionic, electronic, orientation, and charge spacing polarization;

1. As the charge carriers are not able to rotate rapidly enough when the frequency of the applied field increases, their oscillation will fall behind the field causing a decrease in the dielectric constant.
2. The inability of the dipole to rotate and hence its inability to follow the total field leads to a stop in the orientation polarization, which in turn is reflected on the dielectric constant leading to its decrease.
3. Finally, the space charges polarization also leads to a decrease in the dielectric constant.

For all studied temperatures and frequencies, diminution of the dielectric constant was observed up to 2 mol % of Ni ions followed by an increment starting from 3 mol% of Ni as shown in Fig. 8b. Due to the charge/size ratio of the Ni ions, it may permeate into the glass network as pieces of [NiO_4_]^2-^ in the tetrahedral coordination forming Ni–O-P bond, leading to a decrease in the disorder in the glass network, which results in a decreasing in the dielectric constant. Starting from 3 mol %of nickel, Ni generates bonding defects due to its new role in the glass network as a modifier. These defects pave an easy path for the free charge of conducting species migration intensifying the space charge polarization causing an augmentation in the dielectric constant.

**Figure 9:**
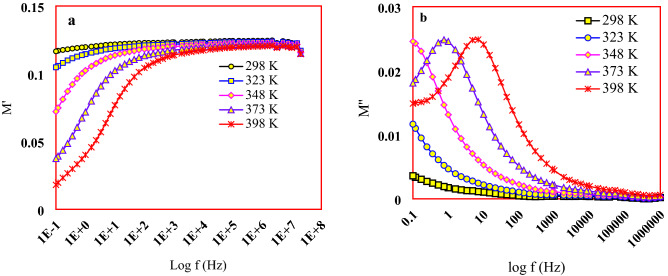
Dependence of (**a**) M' and (**b**) M'' on different temperatures and frequencies.

The electric modulus *M'* approached to the constant value $M_{\infty }$ at low frequency region and the plateau region also appeared in low frequency. The rapidly augmentation of electric modulus *M'* with the raising frequency as shown in Fig. 9a attributed to the short-range mobility of charge carriers. A lack in restoring force of the flow charges is conducted response to the electric field reflecting on the charge carriers’ mobility range causing its shortness. The imaginary part of electric modulus *M''* is shown in Fig. 9b. As the temperature increment, the movement speed of the charge carriers increases leading to an increase in the relaxation rate causing a shift of the frequency peak maximum $f_{max}$ towards the higher frequency.

### Optical properties

**Figure 10:**
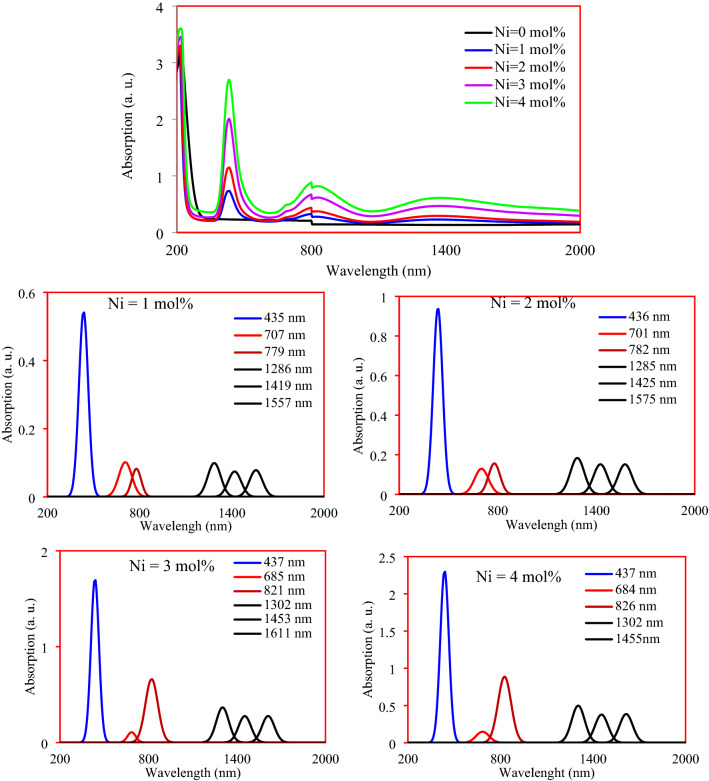
Optical absorption spectra and the deconvolution of the studied samples.

**Figure 11:**
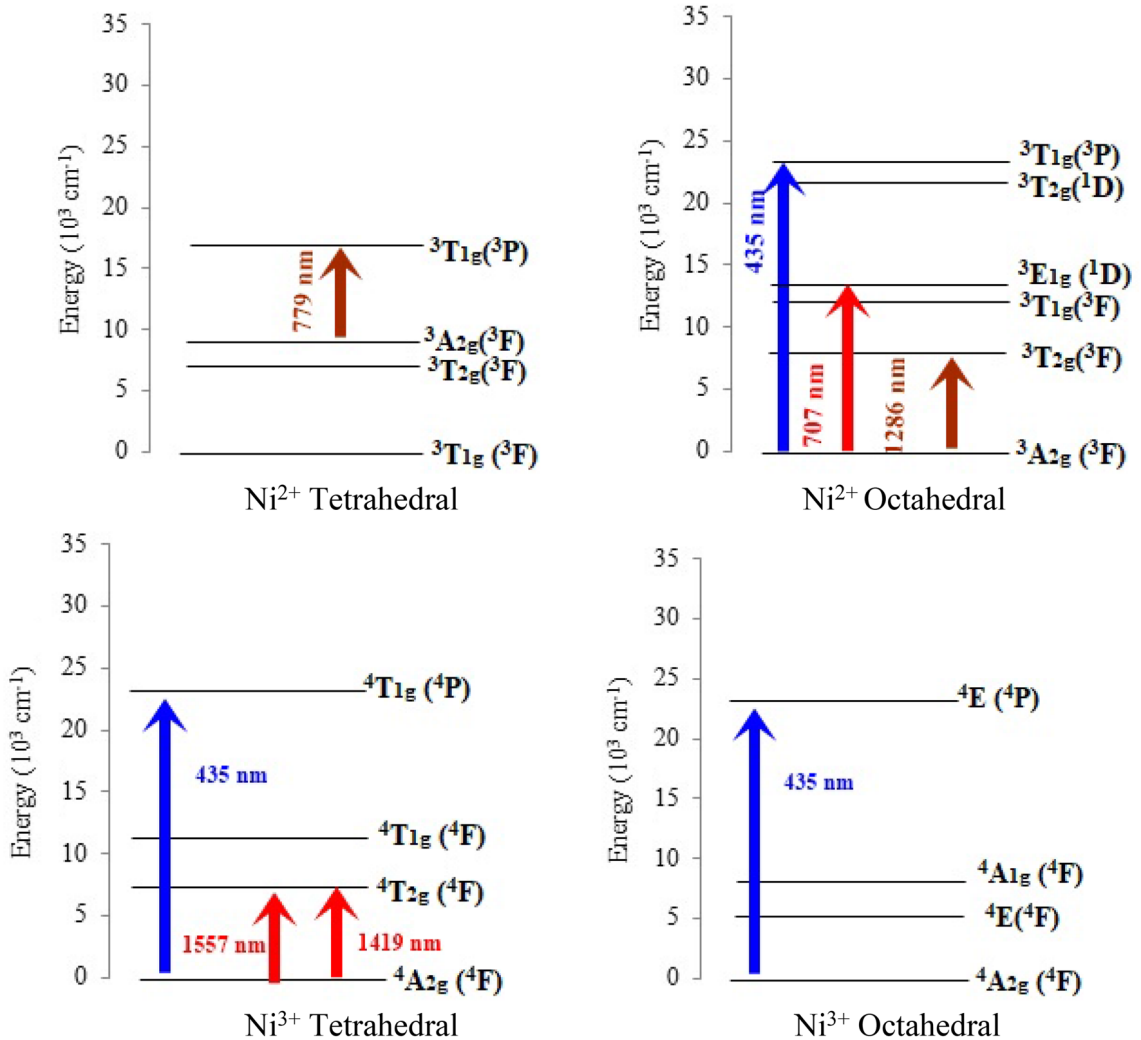
Energy levels diagram and transition of both Ni^2+^ and Ni^3+^ in octahedral and tetrahedral form for the present glasses.

In the optical absorption spectra and their deconvolution shown in the Fig. 10, many absorption bands appeared indicating the presence of both Ni^2+^ and Ni^3+^ ions. The appeared absorption band at 224 in the nickel free specimen is due to the presence of impurities in the raw materials or charge transfer band for ferric ions. Three bands were observed in the absorption spectra of 1 mol% of Ni, one of which is almost sharp, around 435 nm, while two broad bands appeared, the first of which is in the range from 660 to 990 nm and the other from 1150 to 1650 nm. A deconvolution for all Ni-doped samples was conducted to find out the origin and behavior of these domains. In deconvoluted spectrum of 1 mol% of nickel, seven bands appeared at 435, 707, 779, 1286, 1419, and 1557 nm. The occurred transitions in different energy levels inside the octahedral and tetrahedral of both Ni^2+^ and Ni^3+^ ions are shown in Fig. 11. The band located at 435 nm is considered as a superposition of the three optical absorption lines of ^4^A_2g_(^4^F) $\to$ ^4^E(^4^P) electronic transition in the *octahedral* Ni^3+^(3d^7^) ion, ^4^A_2g_(^4^F) $\to$ ^4^T_1g_(^4^P) transition in the *tetrahedral* Ni^3+^ (3d^7^) ion, and the *allowed* ^3^A_2g_(^3^F) $\to$ ^3^T_1g_(^3^P) transition in *octahedral* the Ni^2+^

(3d^8^) ion^24–26^. The band at 707 nm harmonious to the *forbidden transition* ^3^A_2_(^3^F) $\to$ ^3^E_1g_(^1^D) in Ni^2+^ *octahedral*, while that is at 779 nm due to the *allowed* transition ^3^A_2g_(^3^F) $\to$ ^3^T_1g_(^3^F) in Ni^2+^ *tetrahedral*^24–26^. The observed band at 863 nm assigned to ^3^A_2g_(^3^F) $\to$ ^3^T_1g_(^3^F) *allowed* electronic transition in *octahedral* Ni^2+^ ions. The band at 1286 nm is due to ^3^A_2g_(^3^F) $\to$ ^3^T_2g_(^3^F) *allowed* electronic transition in *octahedral* Ni^2+^ ions^27,28^. The Low-intensity broad band, which deconvoluted to two bands at 1419 nm and 1557 nm is assigned to the *allowed* transition ^3^A_2g_(F)$\to$ ^4^T_2g_(^4^F) in Ni^3+^ *tetrahedral*^25,27,28^. The appearance of three distinct bands for N^3+^, especially those in the NIR region 1419 nm and 1557 nm, is in agreement with ESR results. The redshift in the absorption peaks was attributed to the increase in the average bond length of ${\text{Ni}} - {\text{O}}$ with the increase in the penetration of nickel ions. Redshift and increase in both of the characteristic peaks of the octahedral and tetrahedral meant the richness of the studied glass network by both octahedral and tetrahedral for all Ni contents.

Ligand field parameters; crystal field parameters Dq, Racah parameters B &C, nephelauxetic ratio $\beta$, and nephelauxetic function *h* were estimated using the following Eqs. ^29,30^ and tabulated in Table 1

$$D_{q} = \frac{1}{340}\left( {9\nu_{2} + 9\nu_{3} - \sqrt {81\nu_{2}^{2} - 178\nu_{2} \nu_{3} + 81\nu_{3}^{2} } } \right)$$

$$B = \frac{1}{15}\left( {\nu_{3} + \nu_{2} - 30D_{q} } \right)$$

$$C = 4.709 \pm 0.5$$

$$\beta = {\raise0.7ex\hbox{$B$} \!\mathord{\left/ {\vphantom {B {B_{free} }}}\right.\kern-\nulldelimiterspace} \!\lower0.7ex\hbox{${B_{free} }$}}$$

$$h = \frac{1 - \beta }{{k_{{Ni^{2 + } }} }}$$

**Table 1:** Ligand field parameters, optical band gap, and Urbach energy of produced glasses.

Ni Con. (mol%)	$\nu_{2}$(nm)	$\nu_{3}$(nm)	$Dq$(cm^-1^)	B (cm^-1^)	C (cm^-1^)	$Dq/B$	$\beta$	$h$	${\varvec{E}}_{{\varvec{g}}}$(eV)	$\Delta E$(eV)
0	–	–	–	–	–	–	–	–	3.98	0.358
1	779	435	771	846	3986	0.911	0.784	1.802	3.45	0.368
2	782	436	767	847	3988	0.906	0.784	1.797	3.21	0.700
3	821	437	724	890	4189	0.814	0.823	1.500	3.02	0.712
4	826	437	719	895	4213	0.804	0.828	1.430	2.83	0.940

for Ni^2+^ $B_{free} = 1080 cm^{ - 1}$ and $k_{{Ni^{2 + } }} = 0.12$^29,30^

As it is observed from Table 1, both $Dq$ and B parameters were found to follow the well-established opposite behavior, where $Dq$ decreases but B increases with an increase of NiO mol %. These results indicate a strong electron localization at Ni ions and thus, the inter-electronic repulsion in the d shell becomes relatively more intense, resulting in chemical bonds between Ni ions and the ligands with a more ionic character. The nephelauxetic ratio $\beta$ growth confirms the augmentation of electrons localization, i.e. the decrease in the delocalization, which means an increase in the rigidity of the glass network that fully agrees with the mean phosphor—phosphor separation results. This result also confirms the increased ionic character around the Ni ions. The estimated values of $h$ parameter decrease with Ni ion content, mirroring a decrease in the covalent bonding nature between Ni ion and the surrounding ligands as well as increased d-electrons localization. The obtained values for the $Dq/B$ ratio showed that the crystal-field sites in all investigated samples are weak.

**Figure 12:**
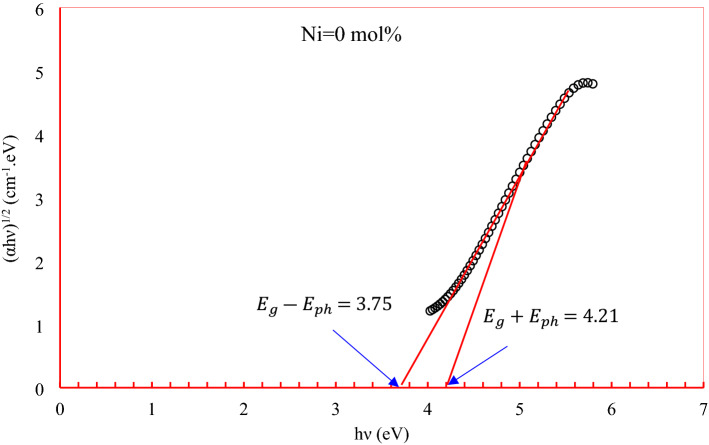
Phonon absorption and emission of Ni doped glasses to calculate optical band gap.

The band gap and Urbach energy results are listed in Table 1. The value of the band gap is obtained through the mechanisms of phonon absorption $E_{g} - E_{ph}$ or emission $E_{g} + E_{ph}$ ($E_{g}$ is the indirect band gap and $E_{ph}$ the phonon absorption) that is stimulated during the photon absorption as shown in Fig. 12^18,31^. While the Urbach energy $\Delta E$ results were calculated through the slope reciprocal resulting from the relationship between the absorption coefficient logarithm $\ln \alpha$ and the photon energy $h\nu$^32–35^. The observed reduction in the optical band gap $E_{g}$ with the rise in the concentration of the Ni ions attributed to the creation of a great number of donor centers; consequently, the excited states of localized electrons originally trapped on Ni^2+^ positions initiate to overlap with the unfilled 3d states on the neighboring impurity sites. As a result, the impurity band becomes more expanded inside the major band gap. The increase of Urbach energy confirms the growth in the disorder inside the produced glass network, thus the presence of NiO in the system has made it easy for the electrons to move through the materials.

**Figure 13:**
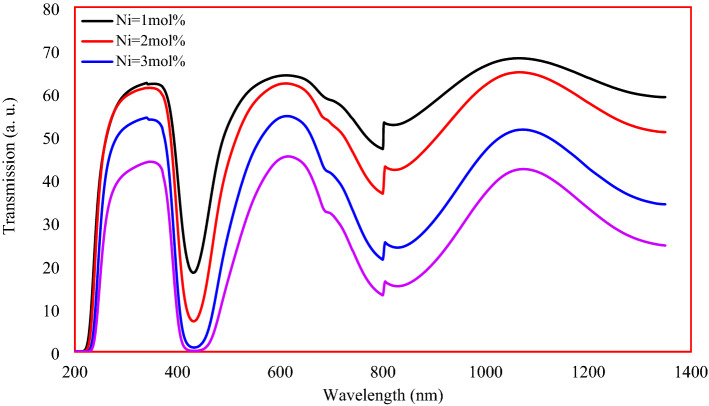
Optical transmittance spectra of the studied samples.

Figure 13 shows the optical transmittance of the studied Ni-doped glass. Three bands in the UV, visible, and NIR appeared in the transmittance spectrum of the 1 mol% of Ni sample. The UV band was centered at 354 nm and has a 127 nm width and a height of 63%. The visible light band centered at 620 nm owned a width of 270 nm and a height of 64%. As for the NIR range, the band was centered at 1074 and has a width of 344 nm and a height of 68%. The appeared small band at 816 nm is attributed to the artifact from the monochromator change of the spectrophotometer^36^ . The transmitted bands' reduction in height with the increase of Ni ions concentration is attributed to the attenuation of light when it interacts with glass. Ni ion is rich in electrons, causing augmentation of an atomic number of the glass, leading to the attenuation of light photons. On the other hand, the bandwidth becomes sharpest with the increase in Ni concentrations, which is attributed to the shrinking of the interionic distances causing rapprochement in the Ni energy levels, making the electron transfer easier and taking less time to occur. The bandpass specifications in the three regions are listed in Table 2. The low selective transmittance band that observed at 804 nm.

**Table 2:** UV, Visible, and NIR bandpass regions characteristics.

Ni ion Conc. mol%	Bandpass region											
	Ultraviolet				Visible				Near infrared			
	Cut off (nm)	Center (nm)	Width (nm)	Height (%)	Cut off (nm)	Center (nm)	Width (nm)	Height (%)	Cut off (nm)	Center (nm)	Width (nm)	Height (%)
1	408	354	127	63	804	620	270	64	1280	1074	344	68
2	408	340	125	61	794	610	241	62	1286	1070	340	65
3	424	338	121	54	798	620	236	55	1320	1076	336	51
4	418	346	118	44	800	628	233	45	1334	1076	330	43

## Conclusion

A broad bandpass optical filter of Ni-doped oxyflourophosphate glass was achieved. Three UV, Visible, and NIR transmittance bands centered at 354, 620, and 1074 nm were characterized for the achieved filter. Nickel insertion imparts a brown color to the proposed glass network. A wide study of structural, thermal, magnetic, electrical, and optical was conducted to determine the role of nickel and its oxidation states. Both Ni^2+^ and Ni^3+^ states in octahedrally and tetrahedrally geometry were found in the produced glasses. Ni played a modifier role in the studied glass, causing its compactness and thermal stability. The results indicated the absolute dominance of the ionic nature of the bonding between Ni ions and ligands. Ni ions augmentation imparts a semiconducting nature to the studied glass, which was evident from the decrease of the optical band gap energy from 3.98 to 2.83 eV. In the same regard, the high temperature enhances the semiconducting nature of the glass, which was evident in the ac conductivity results. Accordingly, the produced glass has a strong property that makes it suitable as a broadband optical bandpass filter.

## Data Availability

The datasets used and/or analyzed during the current study available from the corresponding author on reasonable request.
